# Supplementary material for: Influenza A vs. COVID-19: A Retrospective Comparison of Hospitalized Patients in a Post-Pandemic Setting
Source: Microorganisms. 2025 Aug 6;13(8):1836. doi: 10.3390/microorganisms13081836 (PMC12388448; doi:10.3390/microorganisms13081836)
Supplement: Supplementary file 1 [file microorganisms-13-01836-s001.zip › microorganisms-3763411-supplementary.pdf]

Table S1. Symptoms at presentation of COVID-19 and influenza A patients.

**Variables**	**COVID-19 (*n*=899)**	**Type A Influenza (*n*=423)**	***p*-value**
Fever, *n* (%)	467 (51.95)	288 (68.09)	< 0.001
Cough, *n* (%)	615 (68.41)	372 (87.94)	< 0.001
Dysphagia, *n* (%)	254 (28.25)	90 (21.28)	0.007
Headache, *n* (%)	224 (24.92)	142 (33.57)	0.001
Ocular pain, *n* (%)	5 (0.56)	18 (4.26)	< 0.001
Myalgia, *n* (%)	180 (20.02)	150 (35.46)	< 0.001
Dyspnea, *n* (%)	330 (36.71)	207 (48.94)	< 0.001
Rhinorrhea, *n* (%)	125 (13.9)	54 (12.77)	0.573
Anosmia, *n* (%)	8 (0.89)	1 (0.24)	0.286
Confusion, *n* (%)	62 (6.9)	23 (5.44)	0.313
Diarrhea, *n* (%)	106 (11.79)	57 (13.48)	0.385
Vomiting, *n* (%)	208 (23.14)	114 (26.95)	0.132
Asthenia, *n* (%)	420 (46.72)	174 (41.13)	0.057
Syncope, *n* (%)	106 (11.79)	39 (9.22)	0.163
Influenza-like illness (fever, cough, onset in the past 10 days), *n* (%)	389 (43.27)	259 (61.22)	<0.001

Table S2. Comorbidities and laboratory parameters of patients with fatal outcomes from Omicron COVID-19 and Influenza A.

Variables	COVID-19 *n*=51	Type A Influenza *n*=14	*p*-value
Comorbidities, n (%)			
Active cancer	7 (13.73)	2 (14.29)	1
Asthma	2 (3.92)	0 (0)	1
Atrial fibrillation	17 (33.33)	6 (42.86)	0.54
AIDS	2 (3.92)	0 (0)	1
Connective tissue disease	2 (3.92)	0 (0)	1
Chronic kidney disease	13 (25.49)	3 (21.43)	1
Chronic hepatitis	1 (1.96)	0 (0)	1
Congestive heart failure	18 (35.29)	9 (64.29)	0.051
COPD	6 (11.76)	4 (28.57)	0.203
Dementia	12 (23.53)	3 (21.43)	1
Diabetes mellitus	18 (35.29)	5 (35.71)	1
Hemiplegia	8 (15.69)	3 (21.43)	0.691
Hypertension	43 (84.31)	11 (78.57)	0.691
History of myocardial infarction	6 (11.76)	0 (0)	0.327
History of stroke or TIA	14 (27.45)	8 (57.14)	0.056
Ischemic heart disease	20 (39.22)	7 (50)	0.468
Leukemia	2 (3.92)	0 (0)	1
Lymphoma	1 (1.96)	0 (0)	1
Liver cirrhosis	0 (0)	2 (14.29)	0.044
Obesity	11 (21.57)	4 (28.57)	0.721
Peptic ulcer disease	1 (1.96)	0 (0)	1
Peripheral vascular disease	7 (13.73)	2 (14.29)	1
Pregnant/post-natal	0 (0)	0 (0)	1
SOT recipient	0 (0)	0 (0)	1
Laboratory findings, median (IQR)			
Leucocyte[1]	12.12 (7.09 - 15.7)	9.02 (6.77 - 13.36)	0.405 [n1=48,n2=14]
Neutrophils[1]	9.45 (6.63 - 13.28)	7.1 (5.64 - 11.83)	0.468 [n1=48,n2=14]
Lymphocytes[1]	0.59 (0.36 - 0.96)	0.55 (0.42 - 0.79)	0.866 [n1=48,n2=14]
Monocytes[1]	0.42 (0.27 - 0.82)	0.45 (0.31 - 0.61)	0.973 [n1=48,n2=14]
Thrombocytes[1]	197.5 (140 - 282.75)	135.5 (110.75 - 237.25)	0.091 [n1=48,n2=14]

NLR	13.73 (6.17 - 36.11)	14.37 (9.63 - 18.45)	0.887 [n1=48,n2=14]
dNLR	6.49 (4.45 - 12.57)	7.29 (6.41 - 9.3)	0.901 [n1=48,n2=14]
PLR	331 (187.26 - 660.2)	288.84 (126.64 - 449.2)	0.354 [n1=48,n2=14]
SII	2921.16 (1129.35 - 6210.57)	1492.86 (923 - 3710.6)	0.345 [n1=48,n2=14]
SIRI	7.43 (1.78 - 19.26)	5 (2.58 - 9.78)	0.954 n1=48,n2=14]
MLR	0.66 (0.29 - 1.74)	0.64 (0.41 - 1.12)	0.662 [n1=48,n2=14]
C-reactive protein	14.39 (8.25 - 22.74)	14.02 (3.68 - 19.85)	0.503 [n1=49,n2=14]
Fibrinogen	497.41 (393.42 - 600.03)	411.26 (322.55 - 500.87)	0.057 [n1=47,n2=12]
LDH	392 (287 - 536)	492 (352 - 726.5)	0.238 [n1=45,n2=8]
Creatinine	1.17 (0.83 - 1.92)	1.47 (1.01 - 2.16)	0.655 [n1=49,n2=14]
Radiological appearance, n (%)			
Ground glass opacities	40 (78.43)	5 (35.71)	0.007
Consolidation, unilateral	3 (5.88)	0 (0)	1
Consolidation, bilateral	41 (80.39)	11 (78.57)	1
Interstitial pattern	21 (41.18)	2 (14.29)	0.112

([1] - $\times 10^3$; IQR – interquartile range; NLR – Neutrophils-to-lymphocytes ratio; dNLR – derived neutrophils-to-lymphocytes ratio, neutrophils/[leucocytes-neutrophils]; PLR – platelets-to-lymphocytes ratio; SII – systemic inflammation index, neutrophils×platelets/lymphocytes; SIRI – systemic inflammation response index, neutrophils×monocytes/lymphocytes; MLR – monocytes-to-lymphocytes ratio; LDH – lactate dehydrogenase; Patients exhibited one or more of the radiologic lesions described).

Table S3. Multivariate Regression Models for ICU Admission, CPAP, Intubation and Death OR Adjusted for sex, ACCI, CRP, creatinine, LDH, and NLR. Model without Splines.

**Variable**	**ICU Admission OR (95% CI), p**	**CPAP OR (95% CI), p**	**Intubation OR (95% CI), *p***	**Death OR (95% CI), *p***
COVID-19 vs. Influenza A	0.79 (0.44 - 1.45) *p*=0.443	0.68 (0.41 - 1.16) *p*=0.151	0.84 (0.41 - 1.82) *p*=0.641	1.5 (0.82 – 2.9) *p*=0.204
Sex (Male vs. Female)	1.02 (0.65 - 1.6) *p*=0.939	1.01 (0.67 - 1.5) *p*=0.970	0.72 (0.4 - 1.26) *p*=0.251	0.87 (0.51 – 1.46) *p*=0.593
ACCI ≥ 4	1.62 (0.9 - 3) *p*=0.117	1.25 (0.76 - 2.07) *p*=0.382	2.85 (1.21 - 7.91) *p*=0.026	5.81 (2.31– 19.55) *p*<0.001
CRP ≥ 4.225	2.11 (1.23 - 3.73) *p*=0.008	3.01 (1.89 - 4.92) *p*< 0.001	3.10 (1.45 - 7.44) *p*=0.006	5.86 (2.9- 13.52) *p*<0.001
Creatinine ≥ 0.89	1.23 (0.76 - 2.01), *p*=0.398	1.21 (0.8 - 1.86) *p*=0.372	1.34 (0.73 - 2.55) *p*=0.357	1.92 (1.08 – 3.6) *p*=0.032
LDH ≥ 213	5.77 (3.34 - 10.54) *p*<0.001	3.61 (2.35 - 5.64) *p*< 0.001	5.83 (2.74 - 14.42) *p*< 0.001	
NLR (Neu/Lym) ≥ 4.67	4.29 (2.45 - 7.93), *p*<0.001	2.86 (1.83 - 4.55) *p*< 0.001	3.56 (1.69 - 8.44) *p*=0.002	

ACCI – Age-Adjusted Charlson Comorbidity Index; CRP – C-reactive protein; LDH – Lactate Dehydrogenase; NLR – Neutrophil-to-Lymphocyte Ratio.
